# Excess Mortality Associated with Antimicrobial Drug-Resistant *Salmonella* Typhimurium

**DOI:** 10.3201/eid0805.010267

**Published:** 2002-05

**Authors:** Morten Helms, Pernille Vastrup, Peter Gerner-Smidt, Kåre Mølbak

**Affiliations:** * Statens Serum Institut, Copenhagen, Denmark

**Keywords:** Zoonoses, *Salmonella*, serotype Typhimurium, mortality, drug-resistance, quinolones

## Abstract

In a matched cohort study, we determined the death rates associated with drug resistance in *Salmonella* Typhimurium. We linked data from the Danish Surveillance Registry for Enteric Pathogens with the Civil Registration System and the Danish National Discharge Registry. By survival analysis, the 2-year death rates were compared with a matched sample of the general Danish population, after the data were adjusted for differences in comorbidity. In 2,047 patients with *S.* Typhimurium, 59 deaths were identified. Patients with pansusceptible strains of *S.* Typhimurium were 2.3 times more likely to die 2 years after infection than persons in the general Danish population. Patients infected with strains resistant to ampicillin, chloramphenicol, streptomycin, sulfonamide, and tetracycline were 4.8 times (95% CI 2.2 to 10.2) more likely to die, whereas quinolone resistance was associated with a mortality rate 10.3 times higher than the general population.

Foodborne *Salmonella* infections have become a major problem in most industrialized countries. Of particular concern is the increasing number of infections with antimicrobial drug-resistant *Salmonella*, including the recent emergence of drug-resistant *Salmonella enterica* serotype Typhimurium (*S.* Typhimurium) definitive phage type 104 (DT104). This strain is usually resistant to at least five drugs: ampicillin, chloramphenicol, streptomycin, sulfonamides, and tetracycline (R-type ACSSuT) and has become a predominant *Salmonella* type in many countries, including the United States, United Kingdom, Germany, and France (*1*–*4*). In spite of its rapid international dissemination (*5*) and the fact that antimicrobial drug-resistant *Salmonella* was associated with human infections before the recent spread of DT104, the available data are inconclusive regarding a possible increased virulence of DT104. Whether antimicrobial drug resistance in DT104 contributes to enhanced illness or death is unclear (*5*–*7*). Few studies have addressed the health impact of drug resistance in types of zoonotic *Salmonella* other than DT104 (*8*–*10*), and these studies suggest that drug resistance may be associated with increased illness and death rates.

Excess mortality associated with drug resistance in zoonotic *Salmonella* is difficult to quantify. Death is a relatively rare event and may not occur until months after the initial diagnosis. Furthermore, a number of factors, including chronic and malignant diseases, may contribute to death from salmonellosis. The objective of this study was to determine death associated with antimicrobial drug resistance in *S.* Typhimurium. The study was based on a large, unbiased sample of Danish patients registered in a national database. We linked these data with those in the Danish civil registry, which has complete information about survival status. Furthermore, by completing the data with information from hospital discharge registries, we were able to adjust for comorbidity.

## Materials and Methods

### Surveillance

In Denmark the diagnosis of human *Salmonella* infections is made at Statens Serum Institut (SSI) or at 10 clinical microbiology laboratories. The SSI receives notifications of positive findings as well as isolates from the microbiology laboratories. If a specific *Salmonella* serotype is found more than once from the same person during a period of up to 6 months, only the first positive sample is registered. As a part of this laboratory-based surveillance system, monitoring for antimicrobial resistance in *S.* Typhimurium was initiated in 1995. In 1995 and 1996, a sample of strains was tested, but from 1997 on, all *S.* Typhimurium strains received at SSI were tested for antimicrobial susceptibility. This study included all isolates of *S.* Typhimurium examined from January 1, 1995, through October 31, 1999.

Isolates were tested by tablet diffusion on Danish Blood Agar (SSI Diagnostica, Hillerød, Denmark) with the use of Rosco Neosensitabs (Rosco, Roskilde, Denmark). The panel included 13 drugs from the Danish Integrated Antimicrobial Resistance Monitoring and Research Programme (*11*). Because reduced susceptibility to ciprofloxacin is difficult to detect by the tablet diffusion test, the E-test (Biodisk, Solna, Sweden) was used as well whenever the tablet diffusion test identified nalidixic acid resistance. In this paper, quinolone resistance refers to strains resistant to the first-generation quinolone nalidixic acid (*12*).

### Registry Linkage Study

All live-born children and citizens of Denmark are assigned a personal identification number, uniquely identifying every person the Danish Civil Registration System (*13*). Demographic data, including vital status, marriage status, emigration/immigration, and address of residence, are kept in this Civil Registration System.

The matched cohort study used the data from the Civil Registration System to compare the death rates of patients with culture-confirmed *S.* Typhimurium infections to the death rates of persons in the general Danish population. For each patient, we randomly selected 10 people matched by age, sex, and county of residence. People who were born during the same month and year as the patient and were alive on the date of sample receipt were eligible for the reference group. From the Danish Civil Registration System, we obtained information on vital status, date of change of vital status, (i.e., date of death or emigration) and area of residence (county level) for the patients and the persons included in the reference group.

Data on admissions to hospital and discharge diagnosis were obtained by using the data from the Danish National Patient Registry (*14*) and the Cancer Registry for all persons included in this study, thereby allowing us to control for preexisting illness (comorbidity). Danish National Patient Registry contains data on all patients discharged from non-psychiatric departments since January 1, 1977. Diagnoses and procedures are coded according to the International Classification of Diseases 8 or International Classification of Diseases 10 (from 1993). Diagnoses obtained during 10 years before infection were used to calculate the comorbidity index.

### Statistical Methods

The comorbidity index used the principles described by Charlson et al. (*15*). This index is a sum of severity scores (weights) corresponding to the number and severity of comorbidity conditions. In the first step, we analyzed the data from the background population to calculate the relative rate associated with each of the diagnostic groups summarized in Table 1. These relative rates served as the weights in the further survival analyses. The index was calculated by adding log-transformed weights, thus taking into account multiple hospital discharges. Diagnostic groups associated with a relative mortality rate <1.2 were not included in the models. By comparing this index with the survival analyses, any difference between the death rates of *Salmonella* patients and the general population quantifies excess mortality beyond what is attributable to underlying illness.

**Table 1:** The distribution of comorbidity diagnosis of 2,047 patients with *S.* Typhimurium infection and a sample of the general Danish population of 20,456 persons

Diagnostic group	No. (%) of *S.* Typhimurium patients	No. (%) in the general population	Severity score (weight) in comorbidity index
Lymphoma or leukemia	19 (0.9)	23 (0.1)	3.40
Metastatic cancers	8 (0.4)	19 (0.1)	2.02
Liver disease	10 (0.5)	35 (0.2)	1.97
Tuberculosis	0	13 (0.1)	1.78
Movement disorders and epilepsy	4 (0.2)	40 (0.2)	1.56
Diabetes	44 (2.2)	186 (0.9)	1.50
Renal disease	31 (1.5)	114 (0.6)	1.37
Inflammatory bowel disease	39 (2.4)	66 (1.7)	1.34
Other neurologic diseases^a^	12 (0.6)	76 (0.4)	1.32
Hemoglobin abnormalities	14 (0.7)	62 (0.3)	1.23
Congestive heart failure	22 (1.1)	103 (0.5)	1.22

^a^Neurologic or neuromuscular diseases other than Alzheimer's disease, multiple sclerosis, Parkinson's disease, Huntington's disease, and epilepsy.

To compare mortality rates of *S.* Typhimurium patients with those of the general population, the data were stratified so that each stratum contained 1 patient and 10 persons from the reference group. To control for age, sex, and county of residence, we used conditional proportional hazard regression. Death up to 2 years after infection was determined, after adjusting the data for comorbidity as described. To assess death rates associated with antimicrobial drug resistance, interaction by drug resistance on *Salmonella* deaths was determined. We used the Wald test to test for homogeneity of the rate ratios. The analyses were conducted by the use of the PHREG procedure of the SAS system (Version 6.12, SAS Inst. Inc., Cary, NC). Death rate ratios (RR) are expressed as the relative death rates of patients compared with the matched sample of the general Danish population, and the term “referents” refers to this unexposed matched sample.

## Results

Of 4,075 cases of *S.* Typhimurium infections reported in Denmark from January 1995 to October 1999, the antimicrobial-drug susceptibility was determined in isolates from 2,059 cases, and a successful link to the Civil Registry System was obtained for 2,047 (99.4%). In the period up to 2 years after entry in the study, 59 deaths were identified in *S.* Typhimurium patients and 221 deaths among 20,456 referents. The median age of the 59 persons were 74.1 years (range 18.1 to 90.1). In the first 30 days after entry in the study, the cumulative mortality proportion (Kaplan-Meier estimate) was 0.73% for *S.* Typhimurium patients and 0.04% for the referents (RR 15.4, 95% confidence interval [CI] 6.1 to 39.2). In the period 30 to 720 days after entry, cumulative mortality was 2.75% in *S.* Typhimurium patients and 1.51% in referents (RR 1.8, 95% CI 1.3 to 2.6). On this basis, we used the period 0 to 720 days in the remaining analyses.

Overall, patients with *S.* Typhimurium were 3.0 times (95% CI 2.2 to 4.0) more likely to die than referents in the 2 years following infection. After the data were adjusted for comorbidity, the relative rate was 2.3 (95% CI 1.7 to 3.2). This relative death rate was independent of age (p=0.84).

A total of 631 (30.8%) patients were hospitalized in connection with the *S.* Typhimurium infection. In the reference group, 577 (2.8%) were hospitalized within 60 days of entry. Five of those had gastroenteritis as their primary diagnosis.

Two hundred seventeen (10.6%) of *S.* Typhimurium patients and 954 (4.7%) persons from the referent group had at least one of the diagnoses listed in Table 1, which summarizes the various diagnostic groups and their weights in relation to the comorbidity index. A total of five HIV infections were found, three among patients and two in the reference group. All five were still living at the end of the study.

In the 2,047 strains, 953 (46.6%) were pansusceptible, 1,094 (53.4%) resistant to at least one drug in the panel, and 639 (30.8%) were resistant to at least two drugs. Resistance to sulfonamides was found in 47.3% of the patient isolates, tetracycline in 25.1%, streptomycin in 22.4%, ampicillin in 19.2%, chloramphenicol in 17.0%, kanamycin in 9.6%, quinolone in 4.1%, trimethoprim in 3.0%, gentamicin in 2.2%, and ceftriaxon in 1.4%. No ciprofloxacin-resistant strains were found. The MIC of ciprofloxacin in the quinolone-resistant isolates ranged from 0.06 to 0.38 mg/L (median 0.09 mg/L).

R-type ACSSuT was found in 283 (13.8%) isolates, and patients infected with this type were 6.9 times more likely to die than the general population, compared with a RR of 2.6 in patients with strains of other R-types (p =0.02). Also, chloramphenicol (7.4 vs. 2.4, p=0.003), quinolones (9.9 versus 2.8, p=0.05), and ampicillin (5.1 versus 2.7, p=0.09) were associated with higher death rates in resistant than sensitive strains.

Table 2 shows the relative death rate associated with antimicrobial resistance after the data was adjusted for coexisting diseases. Infections with pansusceptible strains were 2.3 times (95% CI 1.5 to 3.5) more likely to die than the general population, whereas infection with R-type ACSSuT was associated with 4.8 times (95% CI 2.2 to 10.5) higher mortality. Patients infected with quinolone-resistant strains (R-type Nx) were 10.3 times (95% CI 2.8 to 37.8) more likely to die, and R-type ACSSuTNx was associated with 13.1 times (95% CI 3.3 to 51.9) higher mortality. Three other antimicrobial drugs (trimethoprim, gentamicin, and ceftriaxone) were examined, but because of a low number of resistant strains, valid statistical inference could not be carried out. All the strains resistant to these drugs exhibited R-type ACSSuT. Most (82%) of the chloramphenicol-resistant strains and 72% of the ampicillin-resistant strains were also R-type ACSSuT.

**Table 2:** Two-year relative death rate of patients infected with *Salmonella* Typhimurium, by antimicrobial susceptibility pattern. Registry linkage study including 2,047 patients and a random matched sample of 20,456 people from the Danish general population

	Resistant		Susceptible^a^		
	Deaths/cases	RR^b^ (95% CI^c^)	Deaths/cases	RR (95% CI)	p-value^d^
Resistant to >1 drug	31/1,094	2.4 (1.6–3.7)	28/953	2.3 (1.5-3.5)	0.86
Ampicillin	13/393	3.5 (1.7-7.2)	46/1,654	2.1 (1.5-3.0)	0.21
Chloramphenicol	16/347	5.1 (2.6-10.2)	43/1,700	1.9 (1.4-2.8)	0.01
Streptomycin	13/458	2.1 (1.1-4.1)	46/1,589	2.4 (1.7-3.4)	0.76
Sulfonamides	31/969	2.5 (1.6-3.9)	28/1,078	2.1 (1.4-3.3)	0.60
Tetracycline	15/513	2.2 (1.2-4.1)	44/1,534	2.4 (1.7-3.4)	0.85
Kanamycin	3/108		23/1,018	3.9 (2.2-6.7)	
Quinolone	5/83	10.3(2.8-37.8)	54/1,964	2.1 (1.6-3.0)	0.02
R-type ACSSuT	12/283	4.8 (2.2-10.5)	47/1,764	2.1 (1.5-2.9)	0.06
R-type ACSSuTNx	5/40	13.1 (3.3-51.9)	7/243^e^	2.9 (1.1-7.9)	0.09

^a^Susceptible refers to strains susceptible to the given antimicrobial drugs or combination of antimicrobial drugs; first cell refers to pansusceptible strains.  ^b^Death rate relative to the general population, as estimated by conditional proportional hazards regression analysis, controlling for underlying illness; RR=rate ratio. ^c^CI=confidence interval. ^d^p-value for test of homogeneity, i.e., RR for resistant being the same as for susceptible strains. ^e^Strains with R-type ACSSuT, but quinolone sensitve.

A total of 270 of the isolates with R-type ACSSuT were phage-typed, and 217 (80.4%) were DT104, 18 (6.7%) DT12, 11 (4.1%) DT120, and the rest were other or unknown phage types. Strains with other R-types were distributed over a number of different phage types. A total of 1,667 were examined, and the three most common were DT12 (46.8%), DT66 (6.0%), and U288 (4.9%). Thirty-nine (2.3%) were DT104. In the patients with R-type ACSSuT, no difference in the death rate between persons infected with DT104 (relative death rate 4.4, 95% CI 1.7 to 11.6) and other phage types (relative death rate 6.4, 95% CI 1.3 to 32.4) was found; both estimates were adjusted for comorbidity.

No difference in age and sex distribution between patients infected with R-type ACSSuT and other antibiograms were found. The median age in both groups was 33 years (range 1 to 87 and 0 to 95, respectively, p=0.89).

Finally, we analyzed a model with three levels of resistance: non-ACSSuT, R-type ACSSuT (Nx-sensitive), and R-type ACSSuTNx. The figure shows the survival curve of the referents and patients according to these three groups. In the group of 40 cases with R-type ACSSuTNx, we identified five deaths within the 2-year period after infection, one of those within the first month of infection, three within 6 months, and one within 18 months. The relative risk associated with an infection with R-type ACSSuTNx was 12.4 without adjusting the data for comorbidity. After adjustment, the RR associated with this resistance pattern was 13.1. The median age in this group was 43 years (range 1 to 89), 10 years higher than the R-type ACSSuT quinolone-sensitive group.

**Figure:**
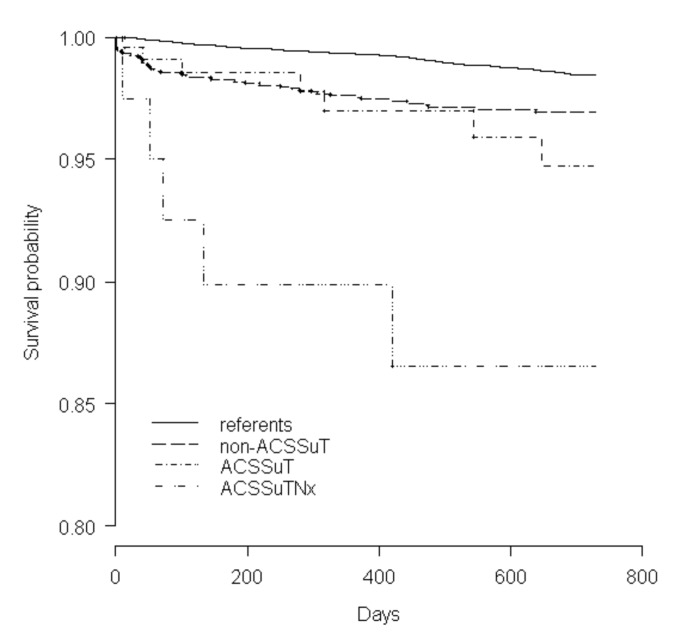
Survival comparison of patients infected with *Salmonella* Typhimurium (by resistance level) to referents. The patients and referents were matched by age, gender, and county of residence.

## Discussion

Since the 1990s, the frequency of antimicrobial drug resistance in zoonotic *Salmonella* and the number of drugs to which the strains are resistant has increased, primarily as a consequence of antimicrobial use in food production (*1*,*9*,*16*–*18*). The recent development of fluoroquinolone resistance is of particular concern (*16*–*21*). At present, a fluoroquinolone is the drug of first choice for extraintestinal and serious intestinal *Salmonella* infections in adults, and resistance to this drug may potentially reduce the efficacy of early empirical treatment. The health impact of antimicrobial drug resistance in zoonotic *Salmonella* needs to be determined (*21*,*22*). We used data from registries created for other purposes to avoid bias and were able to explore long-term death rates and adjust the data for comorbidity.

The comorbidity index was based on discharge diagnoses from patients admitted to hospitals in Denmark and to a lesser degree on data from outpatient clinics but did not include data from general practitioners. Any patient with a coexisting disease severe enough to alter the outcome of a *Salmonella* infection is likely to have had contact with a hospital or an outpatient clinic within the 10-year period before infection. The backbone for the construction of the comorbidity index was the National Discharge Registry. A validation of this registry showed that there was agreement between the registry and hospital records of 75% to 90%, using 3-digit level International Classification of Diseases diagnoses (*14*).

In general, patients with *S.* Typhimurium infections were 2.3 times more likely to die than the matched sample of the Danish population during a 2-year follow-up. This figure is likely to reflect both long-term consequences of *S.* Typhimurium as well as underlying diseases and conditions not fully described by our comorbidity score based on hospital discharge diagnosis. The excess mortality was independent of age, a finding which warrants further studies. The cumulative mortality in the first 30 days, 0.7%, is comparable with the case-fatality rate of 0.8% for all nontyphoidal *Salmonella* serotypes found in data from FoodNet 1996-97 (*23*).

We found that *S.* Typhimurium with R-type ACSSuT was associated with higher death rates than other strains. Similar tendencies were found for chloramphenicol and ampicillin, both being markers for R-type ACSSuT. Patients infected with R-type ACSSuT were seven times more likely to die than the general population, but when the data were adjusted for underlying illness, this figure was reduced to fivefold higher mortality. This reduction was expected; a part of the excess mortality associated with R-type ACSSuT was attributable to underlying illness. However, the excess mortality still tended to be elevated after adjustment. Patients with quinolone-resistant strains had a marked and substantial excess mortality, which could not be explained by imbalances in comorbidity. All the quinolone-resistant strains in this study were designated as fluoroquinolone-susceptible by NCCLS cut-offs for ciprofloxacin. Several patients in the study were part of an outbreak of *S.* Typhimurium DT104 R-type ACSSuTNx traced back to swine herds in the Danish island of Zealand (*17*).

Most deaths occurred in relation to infections with *S.* Typhimurium DT104, and we were not able to demonstrate any statistically significant variation among different phage types. In our initial model we took age into account, expecting a relatively higher mortality among the elderly. But again, we could not demonstrate such an effect. In other words, no additive effect was found between age and drug resistance compared with age and being infected by sensitive strains of *S.* Typhimurium.

A study from England suggests that the isolation rates of drug-resistant DT104 from blood cultures are not higher than those of other *S.* Typhimurium phage types and that the frequency is comparable with the incidence of blood culture isolates of *Salmonella* Enteritidis (*7*). The study suggests that *S.* Typhimurium of R-type ACSSuT does not cause invasive disease more often than *Salmonella* Enteritidis. However, the overall mortality in relation to *S.* Typhimurium infection is higher. Two studies based on outbreaks of resistant *Salmonella* in the United States and the United Kingdom have found case fatality rates of 4.2% and 3.0% respectively (*6*,*8*). Even though they were based on outbreak investigations, the cumulative death rate is comparable to our results (2.9% after 6 months of infection).

Antimicrobial drug resistance in zoonotic *Salmonella* may be associated with adverse consequences in several ways, including treatment failures. However, treatment failures have, until now, been infrequently reported (*17*,*21*). We had no data on treatment with antimicrobial drugs. Therefore, exploring the extent to which the excess mortality of patients infected with quinolone-resistant strains was caused by reduced efficacy of drugs was impossible. We estimate that approximately 20% of the patients were prescribed empiric treatment in connection with the collection of specimens and that some of the deaths may have been associated with reduced efficacy of flouroquinolones, as described in Mølbak et al. (*17*).

Resistant bacteria have a selective advantage in ecosystems where antimicrobial drugs are used. Studies have shown that treatment with antimicrobial drugs (for any reason) is a major risk factor for infections with antimicrobial drug-resistant bacteria, and that this association may result in increased incidence and illness severity (*9*,*24*,*25*). Infection with drug-resistant *S*. Typhimurium in patients treated for other infections may contribute to the excess mortality we found.

Infections with resistant *Salmonella* may be associated with increased severity for reasons that are poorly understood. An increased virulence of drug-resistant *Salmonella* has not been well characterized. Two earlier studies found increased rates of hospitalizations (*10*) and death (*8*), but these studies had limitations. Lee et al. (*10*) were only able to control for comorbidity in a limited way, and none of the earlier studies were restricted to a single serotype and able explore the impact of specific resistance patterns as we did.

The use of antimicrobial drugs in food production is one of the major factors in the emergence and dissemination of antimicrobial drug-resistance in foodborne bacterial pathogens. We were able to determine death rates in a large sample of patients with *S.* Typhimurium and to control for confounding factors in the analyses. We associated resistance in *S.* Typhimurium with excess mortality, and the demonstration of a hazard to human health underscores the need for restrictions in the use of antimicrobial drugs in the production of food from animals. A particular risk was associated with quinolone resistance, indicating that the use of fluoroquinolones for food production animals should be discontinued.
